# Cross talk between miR-214 and PTEN attenuates glomerular hypertrophy under diabetic conditions

**DOI:** 10.1038/srep31506

**Published:** 2016-08-23

**Authors:** Xiaoxia Wang, E. Shen, Yanzhe Wang, Junhui Li, Dongsheng Cheng, Yuqiang Chen, Dingkun Gui, Niansong Wang

**Affiliations:** 1 Department of Nephrology, Tong Ren Hospital, Shanghai Jiao Tong University School of Medicine, Shanghai 200336, China; 2 Department of Nephrology and Rheumatology, Shanghai Jiao Tong University Affiliated Sixth People’s Hospital, Shanghai 200233, P. R. China; 3 Department of Ultrasound in Medicine, Tong Ren Hospital, Shanghai 200336, P. R. China

## Abstract

Glomerular mesangial cells (MCs) hypertrophy is one of the earliest pathological abnormalities in diabetic nephropathy (DN), which correlates with eventual glomerulosclerosis. This study aimed to investigate the therapeutic role of miRNA in diabetic glomerular MCs hypertrophy and synthesis of extracellular matrix (ECM). Microarray analysis revealed a significant up-regulation of miR-214 in the renal cortex of diabetic db/db mice, which was confirmed by real-time PCR of isolated glomeruli and primary cultured human MCs. *In vitro* studies showed that inhibition of miR-214 significantly reduced expression of α-SMA, SM22 and collagen IV, and partially restored phosphatase and tensin homolog (PTEN) protein level in high glucose-stimulated human MCs. Furthermore, we identified PTEN as the target of miR-214 by a luciferase assay in HEK293 cells. Moreover, overexpression of PTEN ameliorated miR-214-mediated diabetic MC hypertrophy while knockdown of PTEN mimicked the MC hypertrophy. *In vivo* study further confirmed that inhibition of miR-214 significantly decreased the expression of SM22, α-SMA and collagen IV, partially restored PTEN level, and attenuated albuminuria and mesangial expansion in db/db mice. In conclusion, cross talk between miR-214 and PTEN attenuated glomerular hypertrophy under diabetic conditions *in vivo* and *in vitro*. Therefore, miR-214 may represent a novel therapeutic target for DN.

Diabetic nephropathy (DN) is one of the most common causes of end-stage renal diseases^1^. Although strict control of blood pressure and blood glucose markedly delay the progression of DN in patients with diabetes, it is very difficult to maintain the intensive therapy and may increase the risk of hypoglycemia^2^. Thus, there is a pressing need for the development of novel and effective approaches for treatment of DN. It is generally accepted that glomerular hypertrophy, mesangial expansion and extracellular matrix (ECM) accumulation are important features of DN, which reduces the area for filtration and leads eventually to progressive loss of renal function^3^,^4^,^5^. Mesangial cells (MCs) hypertrophy is one of the earliest pathological abnormalities in DN, which is involved in the eventual glomerulosclerosis^6^. The MCs hypertrophic phenotype displays characteristics of fibroblast/myofibroblast with an ability to overexpress smooth muscle alpha-actin (α-SMA), SM22, and ECM proteins such as collagens and fibronectin^5^,^7^. Excessive deposition of ECM proteins in the glomerular mesangial area results in mesangial expansion and eventual glomerulosclerosis^8^.

MicroRNAs (miRNAs) are highly conserved small non-coding RNAs, which serve as a unique regulator of gene expression at the posttranscriptional level by directly interacting with the 3′UTR of their target genes. There is increasing evidence demonstrating that miRNAs are involved in a variety of cellular biological functions, such as differentiation, proliferation and metastasis, and so on^9^,^10^. It has been reported that miRNAs play an important role in regulation of hypertrophy of cardiac myocytes and smooth muscle cells^11^,^12^,^13^. The emerging roles of miRNAs in the pathophysiology of kidney diseases were highlighted^14^. It is becoming clearer that a number of miRNAs have been associated with DN and the use of miRNAs holds much promise as a novel treatment^15^. Accumulating evidence suggests that miRNAs play crucial roles in the direct regulation of the ECM synthesis and gene expression during MCs hypertrophy under diabetic condition^16^,^17^,^18^,^19^,^20^,^21^. Moreover, miR-200b/c is involved in glomerular mesangial hypertrophy in the progression of DN^22^. Therefore, miRNAs were recognized to be involved in the development of DN, and targeting inhibition of miRNAs may provide new therapeutic approaches for DN. However, the inhibitory effects of miRNA on MCs hypertrophy and synthesis of ECM in diabetes have not yet been fully investigated. This study aimed to determine whether inhibition of miRNA can ameliorate glomerular MCs hypertrophy and ECM synthesis under diabetic conditions, and then provide a potential novel therapeutic option for DN.

## Results

### Differential miRNA profiles in kidneys from db/db mice

**Figure 1:**
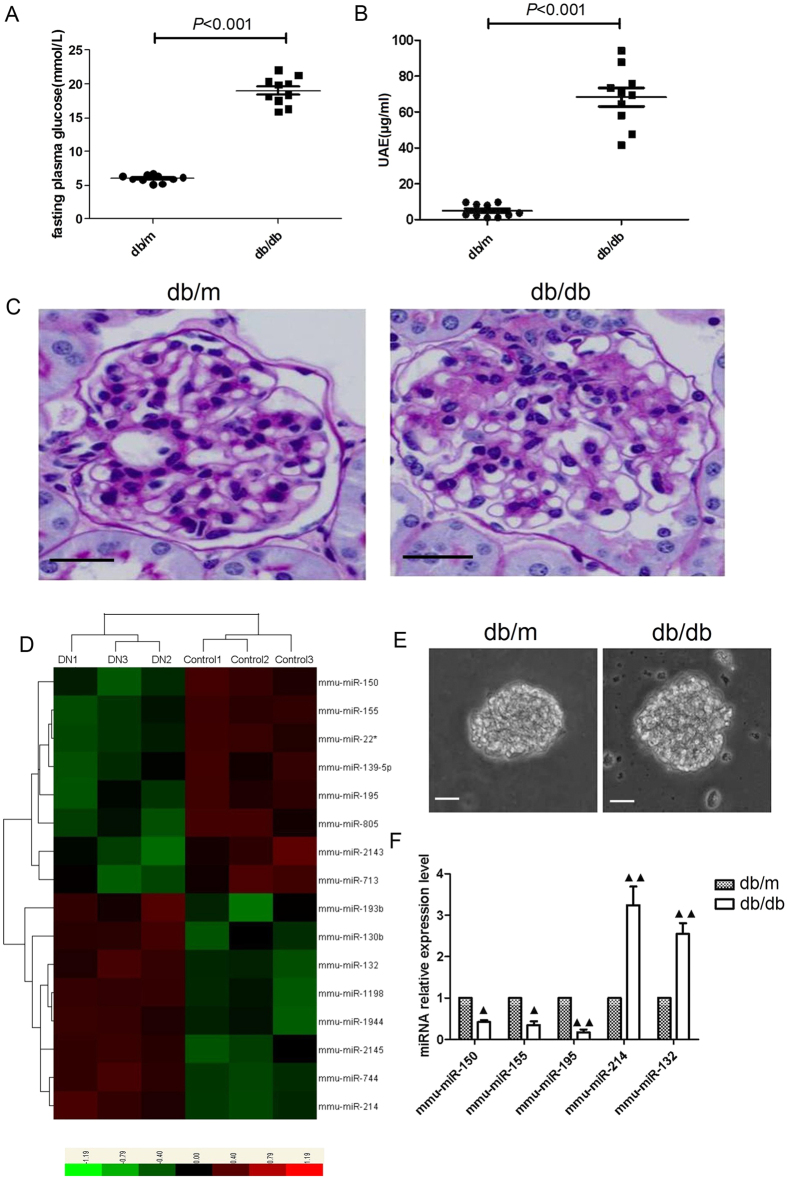
Differential miRNA expression profiles in db/db mice. (**A**) The blood glucose level in mice. (**B**) UAE in mice. (**C**) PAS staining in glomeruli from mice (original magnification ×400). Scale bars: 20 μm. (**D**) Heat map for differential miRNA profiles in renal cortex between db/db and db/m groups. (**E**) Representative images for the isolated glomeruli (Original magnification ×100).Scale bars: 20 μm. (**F**) Quantitative real-time PCR in isolated glomeruli to validate the miRNA data and confirm the gene expression results obtained from microarray analysis. All data were expressed as means ± SD (n = 10/each group). ^▲^p < 0.05, ^▲▲^p < 0.01.

The db/db mice showed higher level of urinary albumin excretion (UAE) and blood glucose when compared with nondiabetic db/m controls (Fig. 1A,B). In addition, glomerular mesangial matrix was increased in db/db mice as compared to non-diabetic db/m controls (Fig. 1C). We then screened the differential miRNA expression profiles in the renal cortex of diabetic db/db mice by miRNA microarray analysis. The heat map for differential miRNA profiles showed that 8 miRNAs were markedly up-regulated, whereas 8 miRNAs were down-regulated in diabetic db/db mice when compared with nondiabetic db/m controls (Fig. 1D). We further performed quantitative real-time PCR in isolated glomeruli to validate the miRNA data and confirmed the gene expression results obtained from microarray analysis. (Fig. 1E,F).

### Bioinformatics analysis of differential miRNAs

**Figure 2:**
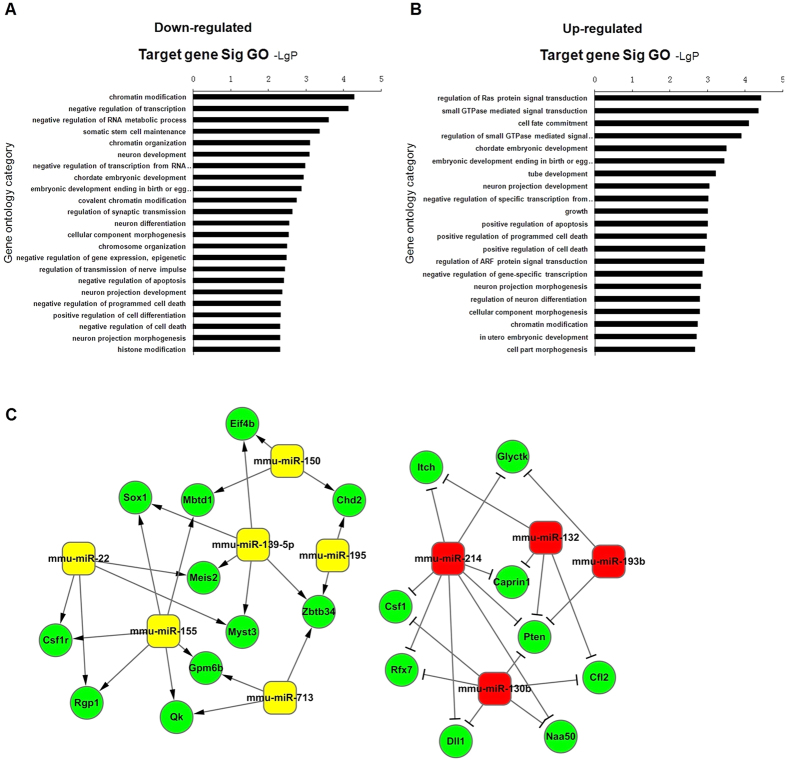
Bioinformatics analysis of differential miRNAs. (**A**) GO analysis of target genes for down-regulation of miRNAs. (**B**) GO analysis of target genes for up-regulation of miRNAs. (**C**) Prediction of miRNA-target gene-network (green circles represent target genes, yellow boxes represent down-regulation of miRNAs and red boxes represent up-regulation of miRNAs, respectively).

To predict potential target genes of differential miRNAs, we integrated the database published online including miRanda IM and TargetScan. A total of 566 target genes were detected for down-regulation of miRNAs, while 446 target genes were detected for up-regulation of miRNAs. Furthermore, we performed Gene Ontology (GO) analysis on all target genes in the David database at http://david.abcc.ncifcrf.gov (Fig. 2A,B). Many important GOs were significantly enriched, among which growth, differentiation and apoptosis were involved in the pathophysiology of DN. The establishment of miRNA-target gene-network was based on the related GOs in Fig. 2C. As shown in supplementary Tables S1 and S2, we used the degree to take miR-214 as the strongest candidate gene among all the candidate miRNAs while PTEN has the highest degree value among all the potential target genes.

### MiR-214 contributed to diabetic MC hypertrophy *in vitro* by targeting PTEN

**Figure 3:**
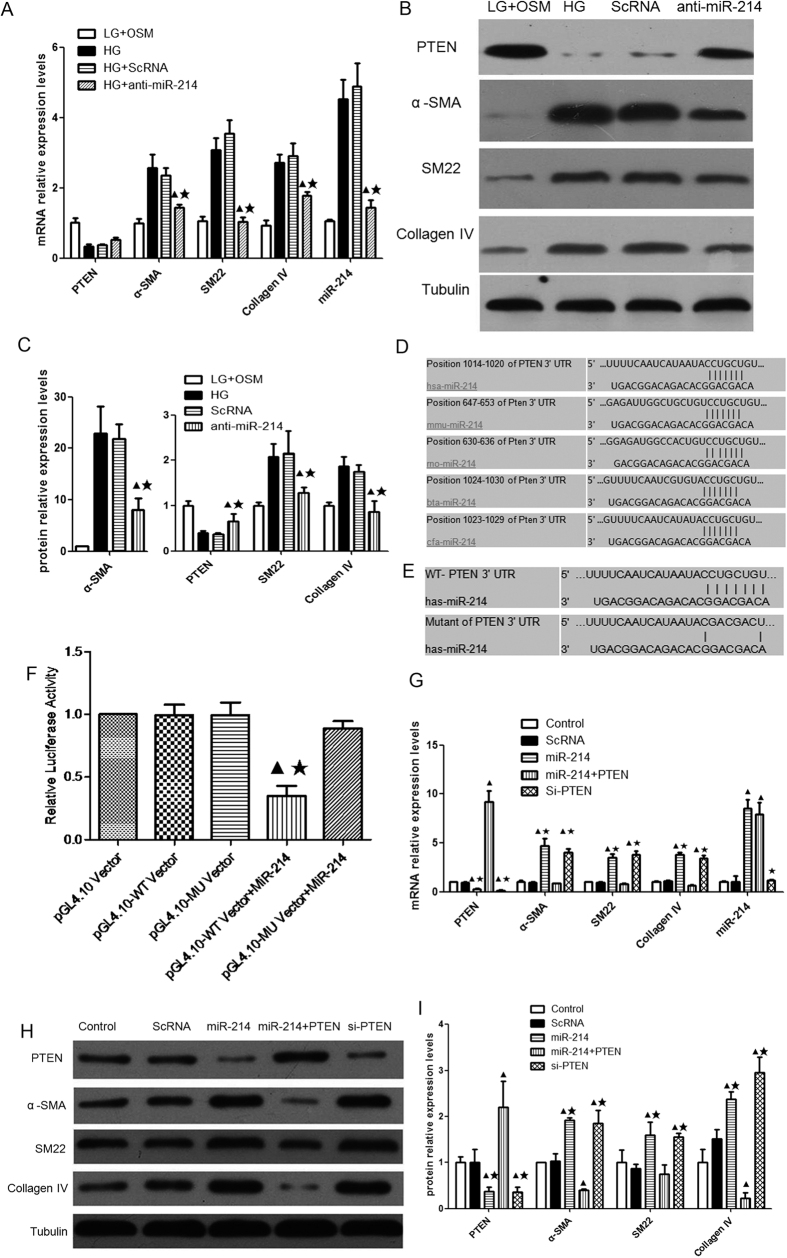
miR-214 contributed to human MC hypertrophy by directly targeting PTEN *in vitro*. (**A**) The mRNA levels of miR-214, PTEN. α-SMA. SM22 and Collagen IV. (**B**) The protein levels of PTEN, α-SMA, SM22 and Collagen IV. (**C**) Quantitative analysis of the protein levels of PTEN, α-SMA,SM22 and Collagen IV. LG + Osm, low glucose (5 mmol/L) supplemented with mannitol (20 mmol/L); HG, high glucose (25 mmol/L); ScRNA, HG with scramble control; anti-miR214, HG with transfection of miR-214 inhibitor. ^▲^p < 0.05 vs. HG, ^★^p < 0.05 vs. ScRNA. (**D**) A genome map for miR-214 binding sites of 3′ UTR of PTEN. (**E**) Schematic diagram indicating the sites of mutant of 3′ UTR of PTEN. (**F**) Dual luciferase measurement performed 48 hours after co-transfection of miR-214 mimics (MiR-214) with wild type (WT) or mutant (MU) of psi-CHECK-2-PTEN-3′UTR vector, respectively, in high glucose-stimulated MCs. ^▲^p < 0.05 vs. pGL4.10 Vector,^★^p < 0.05 vs. pGL4.10-WT Vector. (**G**) The mRNA levels of PTEN, α-SMA, SM22, Collagen IV and miR-214. (**H**) The protein levels of PTEN, α-SMA, SM22 and Collagen IV. (**I**) Quantitative analysis of the protein levels of PTEN, α-SMA, SM22 and Collagen IV. Control: MCs cultured in high glucose served as control; ScRNA: MCs transfected with scramble control; miR-214: MCs infected with lentiviral vectors expressing miR-214; miR-214+PTEN: MCs infected with lentiviral vectors expressing miR-214 and CDS of PTEN. si-PTEN: MCs transfected with siRNA for PTEN. The lentivirus-infected human MCs were cultured in high glucose for 48 hours. All experiments were performed in triplicate. All data were expressed as means ± SD, ^▲^p < 0.05 vs. control, ^★^p < 0.05 vs. miR-214+PTEN.

Transfection of anti-miR-214 reduced the mRNA expression of miR-214, α-SMA, SM22 and collagen IV in high glucose-treated human MCs (Fig. 3A). It also induced a significant decrease in the protein level of α-SMA,SM22 and collagen IV, as well as a significant increase in the protein level of PTEN (Fig. 3B,C). These results indicated that miR-214 contributed to MC hypertrophy, and inhibition of miRNA-214 ameliorated high glucose-induced expression of hypertrophic marker genes and significantly restored PTEN protein level in human MCs.

We performed sequence alignment of PTEN 3′-untranslated region (UTR) by using five species including human, rat, mouse, cow and dog, and then listed miR-214 target sites region (Fig. 3D). We also used a luciferase assay in HEK293 cells to identify whether PTEN as the target of miR-214. We constructed a wild type (WT) or mutated (MU) PTEN-psi-CHECK-2 vector (the mutated complementary sequences of 3′ UTR of PTEN for the seed sequence of miR-214) (Fig. 3E). miR-214 mimics were co-transfected with either WT or mutant-PTEN-psi-CHECK-2 Vector into HEK 293 cells, respectively. The cells were harvested and luciferase reporter activity was measured 48 hours after the co-transfection. The results showed that co-transfection with miR-214 mimics and WT resulted in a significant reduction in luciferase reporter activity compared with the control cells. After mutating the nucleotides of seeding sequence in the 3′ UTR of PTEN, the inhibitory effect of miR-214 mimics on luciferase reporter activity were largely abolished (Fig. 3F). These results demonstrated a direct binding of miR-214 to the 3′ UTR of PTEN.

More importantly, we transfected human MCs with lentiviral vectors expressing miR-214 and coding sequence (CDS) of PTEN in high glucose-treated human MCs to confirm whether miR-214 regulated mesangial hypertrophy by targeting PTEN. The results demonstrated that overexpression of miR-214 inhibited the expression of PTEN and activated the expression of α-SMA, SM22 and collagen IV. In addition, co-transfection of human MCs with lentiviral vectors expressing miR-214 and CDS of PTEN induced an increase in PTEN expression and a decrease in expression of hypertrophic-related genes. In contrast, knock down of PTEN by short interfering RNA (siRNA) in human MCs led to a significant reduction in PTEN protein expression and significant increase in expression of α-SMA, SM22 and collagen IV (Fig. 3G–I). Taken together, overexpression of PTEN markedly attenuated miR-214-mediated MC hypertrophy while knockdown of PTEN mimicked miR-214-mediated MC hypertrophy. Thus, miR-214 regulated mesangial hypertrophy by targeting PTEN.

### Inhibition of miR-214 ameliorated albuminuria and glomerular mesangial expansion in db/db mice

**Figure 4:**
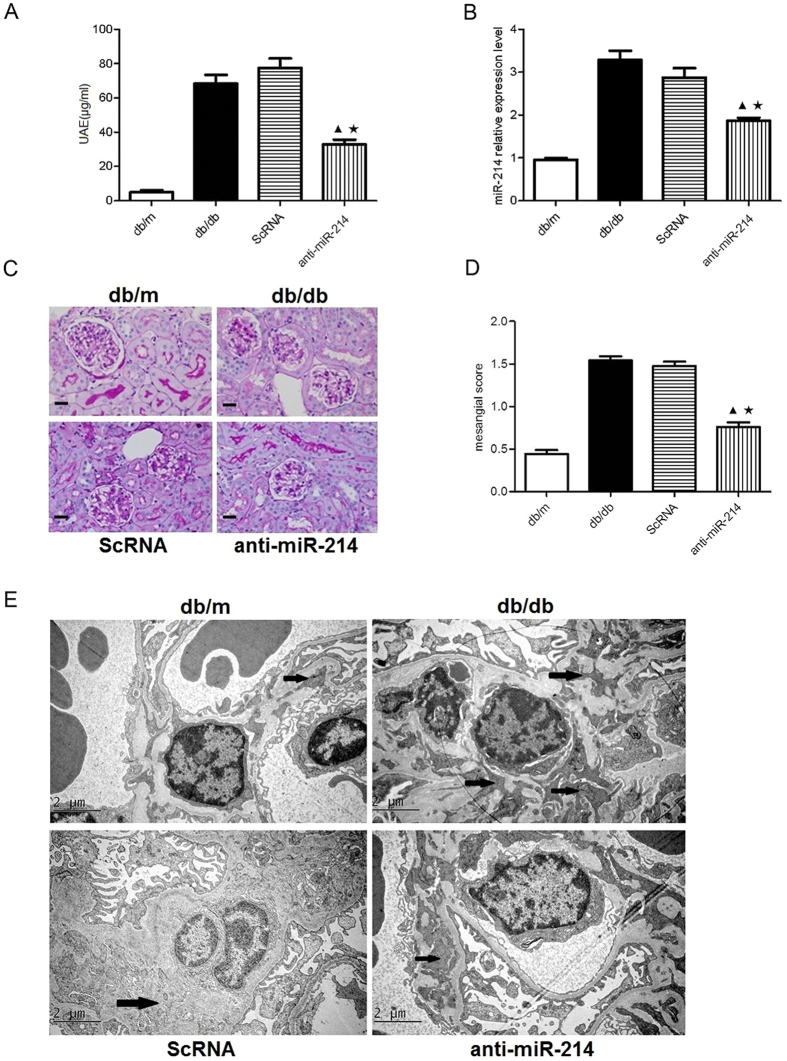
Treatment with miR-214 inhibitor ameliorated the glomerular mesangial expansion in db/db mice. (**A**) UAE in db/db mice. (**B**) mRNA expresion of miR-214 in glomeruli. (**C**) Representative images of PAS staining for glomerular mesangial expansion in mice (original magnification ×400). Scale bars: 20 μm. (**D**) Quantitative analysis of mesangial score in mice. (**E**) Representative electron micrographs in mice (original magnification ×10000). Scale bars: 2 μm. The arrows marked some mesangial areas. db/m, db/m control mice; db/db, diabetic db/db mice; ScRNA, db/db mice treated with miR-214 scramble; anti-miR-214, db/db mice treated with miR-214 inhibitor. All data were expressed as means ± SD, ^▲^p < 0.05 vs. db/db group, ^★^p < 0.05 vs. ScRNA group.

To examine the effect of miR-214 on DN *in vivo*, we delivered lentivirus-packed miR-214 inhibitor at a dose of 1 × 10^7^ TU into diabetic mice by tail vein injections every 2 weeks. At the end of the 12 weeks, UAE was significantly increased in db/db animals compared with nondiabetic db/m mice. However, treatment with miR-214 inhibitor significantly decreased UAE in db/db mice (Fig. 4A). Treatment with miR-214 inhibitor also markedly reduced the miR-214 level in isolated glomeruli from db/db mice. These results indicated that the lentivirus-packed miR-214 inhibitor significantly knocked down the endogenous miR-214 and the delivery procedure was effective (Fig. 4B). Quantitative analysis showed that mesangial expansion scores were significantly increased in db/db mice compared with db/m control animals. In contrast, treatment with miR-214 inhibitor significantly ameliorated mesangial expansion in db/db mice (Fig. 4C,D). Furthermore, we examined the ultrastructure of mesangial area by electron microscopy and found that inhibition of miR-214 also attenuated ECM deposition in db/db mice (Fig. 4E). These results have demonstrated that inhibition of miR-214 significantly ameliorates functional (UAE) and morphological glomerular defects in db/db mice.

### Inhibition of miR-214 attenuated glomerular hypertrophy via targeting PTEN in db/db mice

**Figure 5:**
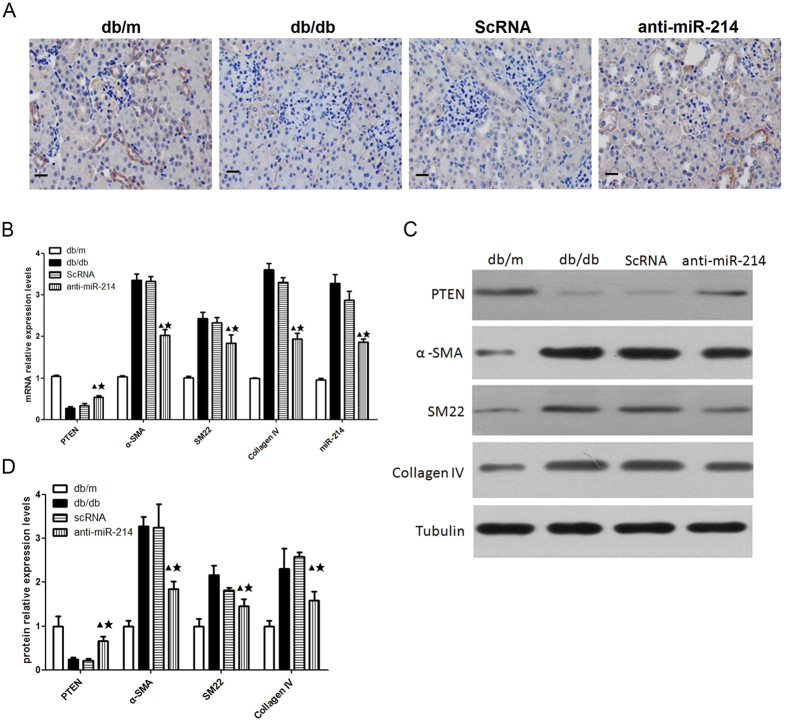
Treatment with miR-214 inhibitor attenuated glomerular hypertrophy, accompanied by restoration of PTEN expression in db/db mice. (**A**) Representative immunohistochemical staining for PTEN in glomeruli (original magnification ×400).Scale bars: 20 μm. (**B**) The mRNA levels of miR-214, PTEN, α-SMA and SM22 in isolated glomeruli detected by quantitative real-time PCR. (**C**) The protein levels of PTEN, α-SMA, SM22 and Collagen IV in isolated glomeruli detected Western blot. (**D**) Quantitative analysis of the protein levels of PTEN, α-SMA, SM22 and Collagen IV. db/m, db/m control mice; db/db, diabetic db/db mice; ScRNA, db/db mice treated with miR-214 scramble; anti-miR-214, db/db mice treated with miR-214 inhibitor. All data were expressed as means ± SD, ^▲^p < 0.05 vs. db/db group, ^★^p < 0.05 vs. ScRNA group.

To further explore the mechanisms by which miR-214 regulated diabetic glomerular hypertrophy, we examined the expression of PTEN in the kidneys. Immunohistochemistry staining revealed that expression of PTEN in diabetic kidneys was significantly decreased compared with that from the control animals (Fig. 5A). In contrast, inhibition of miR-214 significantly restored the expression of PTEN in diabetic kidneys (Fig. 5A). The results of western blot analysis and real-time PCR further confirmed these findings. The mRNA and protein expression of PTEN in diabetic glomeruli was significantly decreased compared with that from the nondiabetic db/m controls. However, inhibition of miR-214 significantly restored the mRNA and protein expression of PTEN in diabetic glomeruli (Fig. 5B–D). Moreover, inhibition of miR-214 also reduced mRNA and protein expression of hypertrophic markers α-SMA, SM22 and collagen IV in diabetic glomeruli (Fig. 5B–D). These data conclusively demonstrated that inhibition of miR-214 ameliorated glomerular hypertrophy in db/db mice and this effect was associated with the restoration of PTEN expression.

## Discussion

Our study demonstrated that cross talk between miR-214 and PTEN attenuated glomerular hypertrophy under diabetic conditions in *vivo* and in *vitro*. This conclusion was based upon the following findings: (i) inhibition of miR-214 ameliorated high glucose-induced expression of hypertrophic marker genes in human MCs; (ii) *in vivo* studies in db/db mice further confirmed that inhibition of miR-214 significantly reduced the expression of SM22, α-SMA and collagen IV, markedly restored PTEN level, and attenuated albuminuria and mesangial expansion; (iii) miR-214 was identified to target PTEN. Overexpression of PTEN could ameliorate miR-214-mediated MC hypertrophy while knockdown of PTEN mimicked the MC hypertrophy. These results strongly indicated that targeting miR-214 might be an attractive strategy to attenuate diabetic kidney injury.

It has been demonstrated that miRNAs are dysregulated in kidney diseases and are thought to contribute to the pathophysiology of the DN^14^. MC hypertrophy precedes marked over-expression of ECM in the diabetic kidneys^6^. MC hypertrophy also triggers synthesis of ECM proteins, and their subsequent deposition in glomeruli, leading to glomerulosclerosis and loss of renal function in DN^5^,^7^. A study showed that miRNA-200b/c was involved in the regulation of glomerular mesangial hypertrophy related to DN^22^. Aberrant expression of miR-214 was identified in a wide range of human tumors such as nasopharyngeal carcinoma, breast cancer, ovarian cancer, colorectal cancer etc., which contributes to the pathogenesis and metastasis of these tumors^23^,^24^,^25^. However, the potential role of miR-214 in the development of DN has not been fully explored. In this study, we showed that miR-214 was markedly upregulated in isolated glomeruli in db/db mice. We also demonstrated that miR-214 promoted human MC hypertrophy and overexpression of collagen IV proteins in the presence of high glucose. However, inhibition of miR-214 significantly reduced expression of hypertrophic markers α-SMA, SM22 and collagen IV, and partially restored PTEN protein level in high glucose-stimulated human MCs. *In vivo* study further confirmed that inhibition of miR-214 markedly downregulated the expression of SM22, α-SMA in isolated glomeruli and attenuated the mesangial expansion in db/db mice. These results clearly demonstrated that inhibition of miR-214 attenuated glomerular hypertrophy under diabetic conditions *in vivo* and *in vitro*.

We further revealed the mechanisms underlying the regulation of miR-214 on MC hypertrophy. First, we listed candidate miRNA genes and target genes on degree level, and used the degree to take miR-214 as the strongest candidate gene and take PTEN as the strongest target gene (data shown in supplementary Tables S1 and S2). Second, we examined the role of miR-214 in the pathogenesis of MC hypertrophy *in vitro*. We found that inhibition of miR-214 reduced the expression of α-SMA and SM22, accompanied by an increase in the protein level of PTEN. Finally, *in vivo* studies further confirmed that treatment with miR-214 inhibitor restored protein and mRNA expression of PTEN in kidney tissue from db/db mice. In addition, our data showed that miR-214 targeted the same sites as previously reported^26^, however, we found that inhibition of miR-214 reduced the expression of SM22, α-SMA, restored PTEN level, as well as attenuated albuminuria and mesangial expansion in db/db mice. We further demonstrated that exogenous PTEN markedly ameliorated miR-214-mediated MC hypertrophy while knockdown of PTEN mimicked the MC hypertrophy. These results suggest that inhibition of miR-214 might ameliorate glomerular hypertrophy under diabetic condition by targeting PTEN.

Inhibition of PTEN contributes to enlargement of cell size in Drosophila and cardiomyocyte hypertrophy in mice^27^,^28^,^29^, whereas hypertrophy of skeletal muscle cells is not detected, suggesting that PTEN functions in a tissue-specific manner^30^. Previous study has demonstrated that expression of PTEN significantly inhibits high glucose-induced protein synthesis and expression of dominant-negative PTEN is sufficient to induce hypertrophy^31^. These results conclusively indicate that PTEN is a key regulator of diabetic mesangial hypertrophy. Our data showed that the expression of PTEN in diabetic glomeruli was significantly decreased compared with that from the control animals. These results demonstrated that hyperglycemia-induced glomerular hypertrophy was associated with a reduction in PTEN expression. Previous study reported that miR-216 and miR-217 promoted TGF β-induced MC hypertrophy *in vitro* by regulating PTEN^32^. We found that miR-214 directly targeted PTEN, which was consistent with PTEN being a target of miR-214 in monocytes *in vitro* as previously reported^26^. To further support our findings, *in vivo* experiments were performed to investigate the role of PTEN as a downstream target of miR-214 in diabetic MC hypertrophy. Our results showed that knockdown of miR-214 significantly attenuated UAE and glomerular mesangial expansion, which was accompanied by restoration of the PTEN level in diabetic db/db mice. Recent study has demonstrated that deletion of miR-214 inhibits tubulointerstitial lesions in a unilateral ureteral obstruction (UUO) mouse model^33^. Our study showed that miR-214 promoted diabetic MC hypertrophy via PTEN and provided an understanding of the role of miRNA in the pathophysiology of DN. In summary, these results suggest that the regulatory effects of miR-214 on PTEN are likely to be accountable for its action against diabetic MC hypertrophy.

A recent study has demonstrated that miR-130 family negatively regulates PTEN protein expression in bladder cancer cells^34^. Our previous study^35^ has demonstrated that miR-196a acts as an important molecular regulator in high glucose-induced MC hypertrophy by targeting p27^kip1^. In this study, we showed that inhibition of miR-214 significantly ameliorated glomerular hypertrophy under diabetic conditions by targeting PTEN *in vivo* and *in vitro*.

There were limitations in this study. First, we only showed changes in hypertrophy markers and did not present the morphology of human MCs at different conditions. Second, podocyte injury is a key event in the initiation and progression of DN. We did not examine the effects of miR-214 inhibition on diabetic podocytes. Thus, the effects of miR-214 inhibition on the morphology of human MCs and podocyte injury in diabetes need to be further investigated in future studies.

In conclusion, cross talk between miR-214 and PTEN attenuated glomerular hypertrophy under diabetic conditions *in vivo* and *in vitro*. These findings suggest that miR-214 may represent a novel therapeutic approach for DN.

## Materials and Methods

### DN animal model in db/db mice with BKS background

Animal care and study protocols were approved by the Animal Ethics Committee of Shanghai Jiao Tong University Affiliated Sixth People’s Hospital, Shanghai, PR China. The License Number of Laboratory Animal was SYXK 2011-0128. All the animal procedures were performed in accordance with the “Guide for the Care and Use of Laboratory Animals” published by the National Institutes of Health. Male six-week-old (40–45 g) db/db mice with background strain C57BKS and heterozygote age-matched db/m mice were purchased from Model Animal Research Center of Nanjing University (Nanjing, China). Mice were kept in the Laboratory Animal Center of Shanghai Jiao Tong University Affiliated Sixth People’s Hospital, Shanghai, PR China. Mice were kept with free water and chow access. All animals were housed in an air-conditioned room on a 12-hour light/ 12-hour dark cycle. Diabetic mice were then randomly divided into three groups (n = 10/each group): (1) untreated db/db group (db/db); (2) db/db group infected with scrambled control and (3) db/db group treated with miR-214 inhibitor (Invitrogen, CA). Age-matched db/m mice were chosen as a control (db/m, n = 10). Lentivirus at a dose of 1 × 10^7^ transfection unit (TU) was delivered to mice by tail-vein injections every 2 weeks. We collected 24 hours of urine to measure urinary albumin excretion (UAE) by indirect competitive ELISA according to the manufacturer’s instructions (Albuwell M; Exocell, PA). The entire experimental period lasted 12 weeks. At the end of the experiments, mice were sacrificed by sodium pentobarbital (2%) intraperitoneal injection. Their left kidneys were then harvested, decapsulated on ice and homogenized by scissors to obtain isolated glomeruli. The isolated glomeruli were stored at −80 °C for real-time PCR and western blot analysis.

### Histological analysis

Renal tissues were fixed in 10% formalin in PBS at room temperature overnight, embedded in paraffin and sliced into 5 μm sections. Periodic acid-Schiff (PAS) staining was performed to assess parameters for glomerular hypertrophy using an Olympus BX51, DP2-BSW microscope as described previously^35^. Briefly, normal glomerulus was scored 0, mild mesangial expansion was scored 1, and moderate mesangial expansion was scored 2. The sections were then examined independently by two blinded investigators.

### Electron microscopy

To determine the morphological changes in glomerular mesangial area, electron microscopic morphometric evaluation was performed by routine procedures. Renal cortex samples were cut into 1 mm^3^ pieces on ice, immediately fixed in 2.5% glutaraldehyde, and then embedded. Ultrathin sections were examined by electron microscopy (Philips CM-120) in a blind fashion. The morphologic assessment was performed.

### Immunohistochemical analysis

Sections of kidneys from the experimental mice were immunostained for PTEN (1:100, Cell Signaling). Immunostaining procedures were performed according to the manufacturer’s instructions. Paraffin-embedded sections (5 μm-thick) were deparaffinized with xylene and rehydrated through a descending ethanol gradient. Primary antibodies were diluted in PBS containing 1% bovine serum albumin (BSA). All antibodies were incubated for 45 minutes at room temperature. Sections incubated with PBS, instead of the primary antibody, served as the negative controls. In each glomerulus, the percentage of positive area within the glomerular area was calculated. All slides were observed independently by two blinded investigators.

### Cell Culture and lentivirus infection

Primary human cell culture was performed as described previously^36^. Experiments involving samples of human origin were approved by the Ethic Committee of Shanghai Jiao Tong University Affiliated Sixth People’s Hospital with informed consent. All experiments were carried out in accordance with relevant guidelines and regulations. Primary human MCs were cultured in Dulbecco’s modified Eagle’s medium (DMEM) supplemented with 20% fetal bovine serum. The third passage of primary human mesangial cells were used in this study. A total of 2 × 10^5^ human mesangial cells were transferred into a six-well dish. At 50% confluency, the cells were serum starved for 24 hours. To test the role of miR-214 in the pathogenesis of MC hypertrophy, we transfected a miR-214 inhibitor (System Bioscience) into cultured human MCs. The cells were infected with miR-214 inhibitor-expressing lentivirus at a dose of 5 × 10^6^ TU as published previously^37^, while the cells cultured in serum-free DMEM with 5 mmol/L of glucose and 20 mmol/L of mannitol served as a control as previously described^38^. Moreover, human MCs were infected with lentiviral vectors expressing miR-214 and coding sequence (CDS) of PTEN, thus inducing overexpression of miR-214 and PTEN in the cells. Human MCs were also transfected with small interfering RNA (siRNA) for PTEN or scrambled control. The lentivirus-infected human MCs were cultured in serum-free DMEM with glucose at a concentration of 25 mmol*/*L for 48 hours, while the cells cultured in high glucose (25 mmol*/*L) served as a control.

### Luciferase activity measurement

The 3′ UTR of human PTEN (Gene ID: 5728) containing complementary sequences for the seed sequence of miR-214 was amplified by PCR and cloned into the psi-CHECK-2 Vector (Promega, WI) (a wild type of psi-CHECK-2-PTEN-3′UTR, WT). A mutant of the 3′UTR with a mutation of complementary sequences for the seed sequence of miR-214 was developed by the QuikChange II Site-Directed Mutagenesis Kit (Stratagene, CA). The sequence alignment of PTEN 3′UTR was perform by using 5 species in mammalian, including human, mouse, rat, cow and dog. For Luciferase reporter measurement, miR-214 mimics (Invitrogen, CA) were co-transfected with WT or mutant of psi-CHECK-2-PTEN-3′UTR vector into HEK293 cell lines (generous gift from Professor Cijiang John He. Mount Sinai School of Medicine, USA) respectively using lipofectamine 2000 (Invitrogen, CA). We detected firefly luciferase activity by dual-luciferase assays kit (Promega, WI) with renilla luciferase activity as an internal control after 48 hours of transfection^39^.

### Quantitative real-time PCR

Total RNA was extracted in isolated glomeruli and human MCs using TRIZOL reagent (Invitrogen, CA) and quantified with ultraviolet spectrophotometer (SmartSpec plus). 2 μg of total RNA was applied to a reverse transcription reaction using reverse transcription kit (Qiagen, Germany), or TaqMan microRNA reverse transcription kit (Exiqon, Denmark). Real-time PCR was performed by SYBR Premix (Takara, Japan) in a LightCycler (Roche, Switzerland). Primer sequences for miRNAs, PTEN, SM22, α-SMA and collage IV were listed in Table 1. Each reaction was amplified in triplicate and ratio results were calculated based on the 2^−ΔΔCT^ method. Gene expression was normalized to β-actin mRNA levels as an endogenous control.

### Western blot analysis

Isolated glomeruli and transfected human MCs were harvested and subjected to a lysis buffer. The lysates were centrifuged at 12,500 × g at 4 °C for 25 minutes. The protein concentration of supernatants was measured using protein analysis kit (Bio-rad, CA). Equal amounts of proteins were immunoblotted with primary rabbit anti-PTEN(1:500, Cell Signaling, MA), SM22, α-SMA and collage IV (1:200, Santa Cruz, TE).The blots were incubated in HRP-conjugated goat anti-rabbit secondary antibody respectively (1:2000, Santa Cruz, TE). The protein signals were visualized by X-rays exposures. All the experiments were performed in triplicate. Protein expression was quantified as the ratio of specific band to Tubulin. Relative protein expression was described as the fold change from the control group.

### Analysis of mi-RNAs microarray and bioinformatics

The miRNA expression profiles of renal cortex from DN animals of db/db mice and controls of db/m mice (n = 3 respectively) was obtained by μParaflo MicroRNA Microarray Assay as described previously^40^. Reported and predicted targets of filtered miRNAs from the Targetscan database (http://www.Targetscan.org/) and miRnaDa database (http://www.microrna.org) were pooled and subjected to GO analysis (http://david.abcc.ncifcrf.gov/). This analysis allows genes to be organized into hierarchical categories, uncovering the miRNA-gene regulatory network on the basis of biological processes as previously established^41^. The interactions between miRNAs and mRNAs were analyzed by their differential expression values, and the network was established according to miRNA-(messenger RNA) mRNA target interactions in Sanger MicroRNA database. The key miRNAs and genes in the network always have the biggest degrees. Therefore, the degree of miRNAs and the target genes was used to get the network of miRNA-mRNA interaction. GO analysis was performed to indicate the miRNA-gene regulatory network. GOs with a p value <0.001 and a false discovery rate (FDR) <0.05 were chosen to calculate the enrichment degree.

### Statistical Analysis

All the data were expressed as means ± standard deviation (SD). Student’s t-test was applied to find whether there was significant difference between the two groups. The significance of the data was determined by ANOVA followed by Dunnett’s multiple range test when necessary. A *P* value < 0.05 was considered statistically significant.

## Additional Information

**How to cite this article**: Wang, X. *et al*. Cross talk between miR-214 and PTEN attenuates glomerular hypertrophy under diabetic conditions. *Sci. Rep.* **6**, 31506; doi: 10.1038/srep31506 (2016).

## Supplementary Material

Supplementary Information
