# Supplementary material for: Cross talk between miR-214 and PTEN attenuates glomerular hypertrophy under diabetic conditions
Source: Sci Rep. 2016 Aug 23;6:31506. doi: 10.1038/srep31506 (PMC4994004; doi:10.1038/srep31506)
Supplement: Supplementary Information [file srep31506-s1.pdf]

Cross talk between miR-214 and PTEN attenuates glomerular hypertrophy under diabetic conditions

Xiaoxia Wang[1,2], E Shen[3], Yanzhe Wang[1], Junhui Li[2], Dongsheng Cheng[2], Yuqiang Chen[2], Dingkun Gui[2] & Niansong Wang[2]

[1]Department of Nephrology, Shanghai Tong Ren Hospital, Shanghai Jiao Tong University School of Medicine, Shanghai 200336, China. [2]Department of Nephrology and Rheumatology, Shanghai Jiao Tong University Affiliated Sixth People's Hospital, Shanghai 200233, P. R. China. [3]Department of Ultrasound in Medicine, Shanghai Tong Ren Hospital, Shanghai 200336, P. R. China

Correspondence and requests for materials should be addressed to D.G. (email:dingkungui@alu.fudan.edu.cn) or N.W (email: wangniansong2012@163.com)

Supplementary information

We listed candidate microRNA genes and target genes on degree level in table form S1 and S2.

Table S1. The degree of miRNAs

microRNAs	degree
mmu-miR-214	8
mmu-miR-155	6
mmu-miR-130b	6
mmu-miR-22	4
mmu-miR-139-5p	4
mmu-miR-132	4
mmu-miR-150	3
mmu-miR-713	3
mmu-miR-195	2
mmu-miR-193b	2

We used the degree to take miR-214 as the strongest candidate gene

Table S2. The degree of target genes

target genes	degree
PTEN	4
Itch	2
Glyctk	2
Csf1	2
Caprin1	2
Rfx7	2
Dll1	2
Naa50	2
Csf1r	2
Rgp1	2
QK	2
Gpm6b	2
Sox1	1
Mbtd1	2
Cfl2	2
Meis2	2
Myst3	2
Eif4b	2
Zbtb34	3
Chd2	2

We used the degree to take PTEN as the strongest target gene
